# Religion, Food Choices, and Demand Seasonality: Evidence from the Ethiopian Milk Market

**DOI:** 10.3390/foods8050167

**Published:** 2019-05-16

**Authors:** Eline D’Haene, Sam Desiere, Marijke D’Haese, Wim Verbeke, Koen Schoors

**Affiliations:** 1 Department of Agricultural Economics, Faculty of Bioscience Engineering, Ghent University, 9000 Ghent, Belgium; Marijke.dhaese@ugent.be (M.D.); wim.verbeke@ugent.be (W.V.); 2 HIVA, Research Institute for Work and Society, University of Leuven, 3000 Leuven, Belgium; sam.desiere@kuleuven.be; 3 Department of General Economics, Faculty of Economics and Business Administration, Ghent University, 9000 Ghent, Belgium; koen.schoors@ugent.be

**Keywords:** milk intake, consumers, demand seasonality, religion, Ethiopia

## Abstract

The impact of religious behavior on food systems in developing economies has been understated in scholarly studies. With its different Christian, Islamic, and traditional faiths, Ethiopia emerges as a suitable country to investigate the impact of religious practices on demand. The inclusion of livestock products in Ethiopian diets is extremely low, even by African standards, a phenomenon often explained by supply and marketing problems combined with low income levels. We deviate from this dominant narrative and single out the impact of religion. We show how fasting practices of Orthodox Christians, the largest religious group, affect milk intake decisions and channels through which consumed milk is sourced. Employing country-wide data collected by the Living Standards Measurement Studies, we find, as expected, that Orthodox fasting adversely affects milk consumption and decreases the share of milk sourced from own production in Orthodox households, an effect we quantify in this paper. Moreover, we observe spillover effects of Orthodox fasting on other religious groups in dominant Orthodox localities. Our findings improve understanding of the broader societal implication of religiously inspired consumption rituals and underscore the challenges resulting from religion-induced demand cycles to design policies that aim at developing the livestock sector.

## 1. Introduction

The term “Livestock Revolution” was introduced in Delgado et al. [1] to point to the unprecedentedly rapid increase in demand for livestock produce noted in the developing world since the 1970s. Population growth, progressive urbanization, and growing per capita income levels are thought to be the revolution’s driving forces [1]. While individual intake of livestock produce indeed exploded in some transforming and urbanized economies of Asia and Latin America (particularly in China, India, and Brazil), the livestock revolution surpassed the majority of developing countries. Annual growth rates of per capita milk, meat, and egg consumption in Sub-Saharan Africa (SSA) were −0.2%, 0.2%, and 0.3% respectively for the period 1987–2007 [2]. Problems of structural supply, poorly developed local markets, low income levels, and lack of consumer awareness regarding the nutritional benefits of animal source foods (ASF) have been advanced to explain this stagnation. Yet, the association of low intake of ASF with cultural values and practices in general, and religion in particular, has been largely neglected and understudied, a research gap we address in this paper. 

The significance of including an appropriate level of ASF in diets to improve dietary quality and nutritional outcomes is underpinned in a number of studies, for example, References [3,4]. ASF are valuable and dense sources of several micro and macronutrients, which are difficult to retrieve in adequate amounts from plant source foods only [5]. Livestock-based interventions are therefore deemed to be a decisive strategy to reduce malnutrition, especially in SSA where half of the world’s malnourished population is residing, and where diets predominantly consist of cereal or root staple crops [6]. Of all livestock produce, milk is the largest source of animal-based protein in developing countries. It accounts for 60% of total per capita consumption of primary livestock products (i.e., meat, milk, and eggs) [2]. In particular, the fact that milk production has one of the lowest production costs when compared to other plant and animal protein sources makes it a desirable source of nutrients for improved nutritional and health outcomes in developing countries [7].

Within SSA, Ethiopia hosts the largest estimated livestock herd [8], yet Ethiopian diets contain relatively little ASF. Animal produce accounts for only 1.7% and 3% of the total energy intake of rural and urban households, respectively [9], whereas starchy staples provide three-quarters of the total calorie intake in Ethiopia [10]. Desiere et al. [11] reported that only 19% of the households interviewed by the Living Standards Measurement Survey (LSMS) in Ethiopia consumed meat or fish. In August 2015, the Ethiopian government launched the national Livestock Master Plan to boost livestock production and productivity. Roadmaps were developed for different key livestock value chains. Although the plan acknowledges the importance of promoting livestock produce consumption, the core of the proposed interventions relates to improving livestock production and productivity through enhanced herd management, genetic resources, feed, and health care [12]. With this one-sided policy focus on supply side factors, the gap between demand and supply of ASF is likely to widen further in the future.

By contrast, our study emphasizes the importance of the demand side of the milk market. Changes in ASF consumption patterns in SSA societies not only depend on changing income levels, but also reflect prolonged cultural processes that are influenced by deeply embedded religious traditions. Robinson and Pozzi [13] point out that particularly cultural and religious values and practices have slowed down the substitution of high value foods such as ASF for starchy staples in African food baskets. To date, consumption-oriented research has mostly ignored or underestimated the role of the diverse cultural and religious traditions in everyday life decisions [14]. 

In terms of faiths, Christianity (63%) and Islam (30%) are the two dominant religious affiliations in SSA [15]. Both religions have been co-existing for centuries and are amongst the fastest growing religious faiths in SSA [16]. Ethiopia, the second most populous country of the continent, is home to Orthodox (44% of the population), Muslim (34%), Protestant (19%), Catholic (1%), and traditional faiths (3%) [17]. The country hosts the second largest community of Orthodox Christians in the world after Russia [18]. The particular fasting rituals are among the main identifying factors of the Orthodox Church. During fasting spells, which are scattered throughout a religious year, Orthodox followers are presumed to pursue a vegan diet, hence to abstain from consuming any animal products. With around half of the Ethiopian population being members of the Ethiopian Orthodox Church (EOC) and with about 180 Orthodox fasting days per year, religion inevitably influences demand for animal produce in the country. Although several studies mention that fasting undeniably impacts livestock consumption patterns as it is causing a seasonal demand cycle [19,20], no study has attempted to quantify the effect of fasting practices on livestock consumption decisions of households at a national level.

This paper aims to evaluate the impact of religion on households’ milk intake decisions and milk sourcing strategies in Ethiopia. In this study, we operationalize religion as the adherence to certain established religious groups, i.e., to which religious community a person affiliates. Other studies proxy religion by both religious affiliation (what a person beliefs) and religiosity (intensity of a person’s beliefs), but we do not elaborate on religiosity or related literature in this manuscript. Using nationally representative data collected by the Living Standards Measurement Survey, we run both Probit and Heckprobit regressions to assess the impact of religion-related variables (religious affiliation, Orthodox fasting, and Orthodox concentration, and their respective interaction terms) on households’ likelihood to consume milk and the probability to source this consumed milk (partially) from own production or from purchase. Based on our findings, we will argue that religious values and practices (along with other factors) lie at the root of the observed low and stationary consumer demand for animal produce in the country and contribute to an intentional demand seasonality. Moreover, the impact of religious rituals is not limited to demand, as the seasonal demand cycle also causes shifts in sourcing channels of consumed milk at the household level. Furthermore, our results will show that these collective fasting rituals not only affect the practicing Orthodox Christian group, but spill over to other religious communities, and in particular to the Ethiopian Muslim community.

Our research contributes to the literature on the economic and societal impact of religion in food systems in four important ways: (1) we study fasting amongst Orthodox Christian households and its impact on milk consumption in an African context, while most research was done on religion and food habits and consumer choices in a Western context within the predominantly studied religion of Islam; (2) we present results from a nationwide study, while other studies are limited to case studies of a few villages or cities; (3) Ethiopia hosts households of different religious affiliations, which allows the setting of a natural experiment to investigate the impact of religious practices; and (4) the study shows the broader societal impact of Orthodox fasting, which is due to important spillover effects on the consumption and sourcing patterns of households across religious affiliations. Our findings should draw the attention of policy makers when designing livestock programs and of the science community interested in the role of religion in food choice. 

## 2. Background, Data, and Methods 

### 2.1. Linking Religion and Food Consumer Behavior 

Research has tried to link habits, norms, and/or customs to various outcomes at household, regional, sectorial, national, and international levels. Cultural factors have been linked to, amongst others, marriage patterns [21], educational attainment [22,23], migration intention [24], sanitation practices [25], fertility rates [26], female labour force participation [27], wage differentials [28], aid allocation [29], service supply [30], resource management [31], technological innovation [32], state governance [33], corruption [34,35], happiness [36], economic growth [37,38], poverty [39], and agricultural productivity [40,41]. How culture in general, and religion in particular, influences food consumption behavior is, however, vastly understudied. A vast body of literature also focuses on how consumer culture influences religion. This is beyond the scope of our study.

You are what you eat; for a long time, people have lived and practiced their religion through food [42,43,44]. Not only food, but consumption choices at large assist in communicating religious identities to others and thus help to mark religious boundaries [45]. Conversely, religion also influences consumers, both directly via explicit instructions laid out in holy texts and/or by religious leaders, and indirectly through beliefs and attitudes established within the larger religious group [46]. Quantitative analyses on the impact of specific religious beliefs and practices on consumer behavior and associated market outcomes remain scarce and scattered [14,47]. 

A large majority of the studies to date unravel behavioral intentions rather than reveal behavioral actions and have a predominant focus on Islamic traditions. Furthermore, the available empirical research is mostly business oriented. These studies are informative for advertising strategies, store and brand image building, product decisions, and establishing (new) market channels. Essoo and Dibb [48] observe that shopping behavior differs across Hindus, Muslims, and Catholics. Fam et al. [49] find that offensiveness, vis-à-vis advertising controversial product groups (like gender-related produce), differs significantly across religious denominations. Some studies give valuable insights that can guide the establishment of particular religiously guided market channels, e.g., Verbeke et al. [50] and Heiman et al. [14] who focus on different halal and kosher certification systems and accompanying supply channels. Consumption behavior for products that are linked to religious practices is also mirrored by demand differences towards specific products and product characteristics, e.g., fairtrade products [51], alcohol consumption [52], and whole fresh chicken versus cut or frozen chicken [53]. While the diverse range of topics reflected in the literature shows the desire of researchers to gain insight into traditional values and religious patterns from the point of view of marketing and consumer communication, it also shows that the broad impact of religion in shaping individual and collective food consumption choices has been understated in scholarly studies [14]. Rather than focusing on the narrow perspective of religious food specifications, we intend to improve our understanding of religiously inspired consumption rituals and their broader societal implications. 

### 2.2. Religious Food Restrictions

Rules and restrictions are inherent to religious institutions and often relate to dietary practices. These food restrictions give rise to distinct consumption patterns and are established either temporarily (such as fasting) and/or permanently (e.g., restriction to kosher and halal food in Jewish and Islamic traditions, respectively), and require either abstinence from all (e.g., Ramadan from dawn to sunset) or certain foods (e.g., by the abstinence from ASF for Orthodox Christians) [54].

Fasting is a fundamental pillar of several religious denominations. Various studies analyze how participation in Ramadan, the Islamic fasting month, affects outcomes, such as dietary and nutrient intake [55,56], mental and physical health [57,58], educational performance [59,60], physical performance [61,62], foetal development [59,63], and output growth [64].

The existing literature on Orthodox Christians mainly concentrates on the Greek community and examines how religious fasting impacts physical and biochemical body parameters through changing food and nutrient intake patterns [65,66,67]. Only one previous study directly addresses the fasting practice of the Orthodox Church in Ethiopia. Knutsson and Selinus [68] elaborate on Easter fasting and how it impacts food intake amongst families and factory workers in the capital, Addis Ababa, and one village in the Oromia region. They detect a substantial drop in the total volume and quality of protein intake by small children between the age of six months and three years, both in the capital and the village, which can be attributed to the exclusion of milk and other ASF from their diet during Easter fasting. Given the modest appearance of animal produce in non-fasting periods, fasting resulted in a deficiency of 25% compared to the recommended total protein intake, which might be harmful for children’s growth and development. 

Other authors indirectly controlled for Orthodox fasting practices in their studies. Hirvonen et al. [9], who studied seasonality trends in Ethiopian diets, uncovered that the average energy intake and diversity of urban households’ diets sharply fell during the two main Orthodox fasting periods (Easter and Christmas). This trend was not observed in rural areas, possibly because ASF were not as regularly incorporated in rural diets. Other studies mention how Orthodox fasting practices affect ASF purchases, availability, and sales. Negassa [69] evaluates the determinants of purchasing raw milk and butter within two towns of the Oromia region, accommodating 200 urban households. They observe that households who participated in fasting were less likely to purchase raw milk and butter. Moreover, conditional on household’s purchases, the quantity of raw milk purchased declined significantly for households practicing fasting, although the effect of fasting on the quantity of purchased butter was not significant, probably because of the small purchase volumes of butter. Ayenew et al. [70], who studied the supply side of the milk market in peri-urban areas, found that milk sales and prices were significantly higher during non-fasting days, while there was no such effect of fasting versus non-fasting on the volume of butter sales. Only butter prices were slightly higher in non-fasting periods. Both observations imply that the reduced milk sales are not compensated for by an increased sale or consumption of butter. An earlier study by Avery [71] on the meat value chain in Addis Ababa reveals that 85% of the butcheries close on traditional Wednesday and Friday Orthodox fasting days and 43% of the supermarkets report a drop in meat sales on those days. Moreover, the French Vétérinaire Internationale-Institute de l’Elevage [72] found that Orthodox fasting practices put great pressure on abattoirs’ capacity in Addis Ababa as the number of cattle, sheep, and goats slaughtered fell by 75% during the Orthodox Easter fasting period. Such a drop was not observed during the Muslim major fasting season (Ramadan). Finally, Aklilu et al. [73], who examined village poultry consumption and marketing in the Tigray region, established that religious fasts and feasts periodically shift local demand, sales, and prices of poultry. 

In sum, the above-mentioned literature agrees that fasting rituals in Ethiopia have an undeniable impact on demand seasonality in milk and meat markets. This paper builds on these findings by providing a nationwide quantification of the impact of Orthodox fasting and by estimating the spillover effects across religious. 

### 2.3. The Ethiopian Livestock Sector

ASF that are commonly consumed in Ethiopia are dairy products, beef, mutton, goat, chicken, and eggs. Pork is rarely consumed because of religious traditions (Muslim and Orthodox), and also camel, fish, and honey contribute significantly less to the average Ethiopian diet. Dairy products still remain the most important animal component in Ethiopian diets and include a wide variety of products: milk (from cows, camel, goats, and sheep), powdered milk, yoghurt, cottage cheese, buttermilk, and butter. Cow milk and butter are the most important dairy products, representing about half and one-third of total dairy consumption, respectively [19]. Although dairy products are the most important livestock products at the national level, Tafere and Worku [74] found that urban inhabitants consume larger quantities of meat than dairy (11.5 kg of meat per capita annually versus 8.5 kg of dairy products; in rural areas, this ratio is 4 kg of meat versus 18.4 kg of dairy). 

Abegaz et al. [19] found that at the national level, 39% of the consumed ASF are sourced from own production. A large disparity is found between the rural and urban areas in the degree of self-sufficiency; rural areas source about 52% from own production, whereas urban dwellers on average purchase 95% of the ASF they consume. Besides this rural–urban discrepancy, important differences are also found among ASF types. Of all livestock products, the share of dairy products sourced from own production is the highest (about 70% at the national level); 14.5% of the urban dwellers source milk from own production while in rural areas this number rises to about 80%. Having a cow is thus an important way to improve nutritional outcomes, especially so in remote areas where dairy markets are often lacking [75].

Seasonality in the consumption of ASF is widely observed in Ethiopia. Fluctuations in the supply of ASF are partially driving this seasonal consumption, and production variations have been associated with fluctuating livestock product prices [20]. However, religious practices (especially the fasting event within the Orthodox Christian Church) are particularly driving this seasonality in ASF consumption. As such, Abegaz et al. [19] observe that drops in the average value of per capita ASF consumption during the months of March and December coincide with major Orthodox fasting periods (Easter and Christmas fasting, respectively), while peaks in consumption largely overlap with Orthodox festivities.

### 2.4. The Christian Orthodox Church and Fasting Practices

Fasting is a ritual commonly practiced within the Orthodox Church. It is seen as an integral part of religious identity and serves as a measure of piousness [68]. As Boylston [76] witnesses, Ethiopian people would rather ask a person first whether they would fast instead of asking this person whether they are Christian. Unique to EOC (and broad Christian Orthodox Church by extension) is that fasting is performed during different periods throughout a religious year. Orthodox traditions prescribe their members not to eat any food of animal origin. The fasts performed differ in duration and can be categorized into two main types: one day fasts occurring on Wednesdays and Fridays all year round (except for the two months after Ethiopian Easter) and longer fasting seasons around or preceding holy events. The Easter fast, also known as Lent, is the longest continuous and most important of all fasts (55 days). Other major fasting periods occur in December–January (40 days) and August (16 days). Besides commonly imposed fasts, a large variety of spontaneous individual fasts is applied which makes it very difficult to assess the exact number of fasting days. For non-clergy, the number of fasting days amounts to 166 to 180, while priests, nuns, and monks typically fast for about 250 days a year [19,68]. Although fasting rules are strict, pregnant and lactating women, severely ill or weak persons, as well as children below the age of seven can be fully exempt from fasting [68]. 

### 2.5. Study Area and Data 

In this study, we focus on Ethiopia, the second most populous country (105 million inhabitants in 2017) and the fastest growing economy in the African region (GDP growth rate of 10% in 2017). Urbanization is on the rise and Ethiopia is amongst one of the fastest urbanizing countries globally with an annual urbanization growth rate of nearly 5%. Poverty rates were almost cut by half between 2000 and 2015 (from 44.2% of the population living below the national poverty line in 2010 to 23.5% in 2015). Meanwhile, yearly per capita income levels remain low, amounting to US$740 in 2017 and Ethiopia’s human development index ranks 173 out of the 189 countries and territories [77,78]. Regarding Ethiopia’s religious diversity, we reported earlier that the representation of the three major religions—Orthodox Christianity, Islam and Protestantism—is nation-wide on average 44%, 34%, and 19%, respectively [17]. However, concentration of particular religious groups occurs at the regional level. Orthodox Christians are predominant in the Tigray and Amhara regions in northern Ethiopia, while Islam is most prevalent in the eastern Afar and Somali regions. Protestantism is strongest in the Southern Nations, Nationalities and People’s Regional State, and the Gambelia region [17]. 

We use data from the LSMS, a household survey program housed within the Survey Unit of the World Bank’s Development Data Group that provides technical assistance to the Central Statistical Agency of Ethiopia for conducting the actual survey on the territory of Ethiopia. This survey program aims at collecting panel household-level data covering a wide range of topics, though specific focus is on strengthening high-quality agricultural statistics that allow for investigating the role of agriculture in welfare and poverty reduction [79]. The empirical analysis was based on data collected from a total of 5262 households in the period 2013–2014, the second data collection wave. We did not consider the first (2011–2012) or the third (2015–2016) wave as the first wave did not include all urban areas (only rural and small-town areas were surveyed), while the third wave had only 599 observations during Lent across all religious groups, whereas these amounted to 1771 in the period 2013–2014. The data set covers all regional states including the capital, Addis Ababa. Respondents were selected by means of a stratified two-stage cluster sample design [79]. A minimum number of enumeration areas (EAs) was set per regional state, using the Probability Proportional to Size sampling method. The EAs correspond to parts of municipalities or kebeles, the smallest administrative entities in Ethiopia. In total, 433 EAs were covered by the survey, 290 in rural areas, 43 in small town areas, and 100 in major town areas. Within each EA, households were randomly selected. 

The LSMS questionnaire provides information on the individual attributes of household members, including religious affiliation, and household consumption of selected food items using a seven-day recall period. The interviews were implemented between February and April 2014, which was before, during, and after Lent (February 24, 2014–April 19, 2014), the longest continuous fast of the EOC. As we do not have information about households’ actual involvement in fasting rituals, we matched dates of the fasting period with the individual recall period: a household record was labelled fasting if at least part of the seven-day recall period coincided with Lent fasting days, and non-fasting otherwise. This is a reasonable assumption since 87% of the Ethiopian Orthodox Christians report that they fast during holy times such as Lent [80]. We find that a third of the consumption survey records occurred during the Orthodox Lent fasting season. This allowed us to compare consumption patterns outside and during the fasting season. Ramadan took place later that year outside the data collection period (June 28, 2014–July 28, 2014). Detailed consumption data were available for milk but not butter. Moreover, no data were available on the production and/or processing of milk within that seven-day recall period, hence we could only approximate what happens at the market level by looking into the milk sourcing strategies of milk-consuming households.

### 2.6. Econometric Approach

We start from the premise that religious denomination only influences a household’s decision whether to consume milk. We have no reason to expect that, ceteris paribus, religious affiliation as such would influence the level of consumption, conditional on the household consuming milk. Especially during Orthodox fasting, the likelihood to consume animal produce within an Orthodox family diminishes. We allow this effect of fasting on milk consumption to be heterogeneous across religious affiliations by interacting religion and the timing of the consumption records.

As we are only interested in analysing which factors determine a household’s likelihood to consume milk, we applied a Probit regression to model the binary outcome of a household’s decision to consume or not to consume milk:

(1) $$Y_{i,z}=0\;if\;Y^{*}{}_{i,Z}\leq 0$$

(2) $$Y_{i,z}=1\;if\;Y_{i,z}^{*}>0$$

(3) $$Y_{i,z}^{*}=\propto_{0}+\sum^{\text{​}}\propto_{i}R_{i}\;+\sum^{\text{​}}\propto_{i}R_{i}\;F_{i}\;+\;\sum^{\text{​}}\propto_{i}H_{i}^{\prime }+\sum^{\text{​}}\propto_{i}L_{z}^{\prime }+\varepsilon_{i}$$

In the above equations, the dependent variable (*Y_i,z_*) takes a value one if the household consumed milk during the seven days prior to the survey, zero otherwise. The probability that a household *i* in locality *z* chooses an alternative one depends on the net utility (Y^*^_i,z_) a household derives from consuming milk. Only when the net utility of drinking milk is positive will a household decide to consume it. This net utility, in turn, is shaped by a vector of household (*H^’^_i_*) and location (*L^’^_z_*) controls, the household’s religious affiliation (*R_i_*), the interaction term of religious affiliation with fasting (*F_i_*), and the error terms (*ε_i_*). 

As we suspect that the Orthodox fasting practices may also cause shifts in sourcing channels of the consumed milk at the household level, we ran a Heckprobit model in which the first stage (Equation (6)) models the likelihood of a household to consume milk, while the second regression models the probability that (part of) this milk consumption originates from own production or purchase (Equation (7)). The dependent variable *W_i,z_* (or *W^’^_I,z_*) takes a value of one if part of the milk that a household consumed originated from own production or purchase, and zero otherwise. The probability that a household *i* in locality *z* chooses an alternative one depends on the net utility (Y^*^_i,z_) a household derives from consuming milk as well as the net utility a household derives from producing the milk that it consumes (W^*^_i,z_) or from purchasing the milk that it consumes (*W^’^_i,z_*). We assume that the explanatory variables in both the first and the second stage of the Heckprobit regressions are identical, except for household member composition, which in turn is used as an identifying variable in the first stage regression. Vulnerable groups, such as young children and elderly people, are often prioritized and exempt from religious prescriptions when it comes to milk intake at the household level [69,81] and thus will increase the likelihood of a household to consume milk. However, the presence of these groups in the household will not necessarily increase or decrease a household’s probability to source milk from own production, conditional on consuming milk in the first place. We also add the main effect of the density of EOC followers in each enumeration area (*O_ea_*), and its interactions with religion dummies in the second stage regression to study how the propensity to source consumed milk from own production during fasting spells fluctuates across different densities of EOC members. We included these variables based on findings from the descriptive analysis.

(4) $$W_{i,z}\;or\;W^{\prime }{}_{i,z}=0\;if\;Y_{i,z}^{*}>0\;\&\;W_{i,z}^{*}\leq 0$$

(5) $$W_{i,z}\;or\;W^{\prime }{}_{i,z}=1\;if\;Y_{i,z}^{*}>0\;\&\;W_{i,z}^{*}>0$$

(6) $$Y_{i,z}^{*}=\beta_{0}+\sum^{\text{​}}\beta_{i}R_{i}\;+\;\sum^{\text{​}}\beta_{i}R_{i}F_{i}+\;\;\sum^{\text{​}}\beta_{i}H^{\prime }{}_{a,i}+\sum^{\text{​}}\beta_{i}L^{\prime }{}_{z}\;+\varepsilon_{i,1}$$

(7) $$W_{i,z}^{*}\;or\;W^{\prime }{}_{i,z}^{*}=\gamma_{0}+\gamma_{1}O_{ea}+\;\sum^{\text{​}}\gamma_{i}R_{i}+\;\sum^{\text{​}}\gamma_{i}R_{i}F_{i}+\sum^{\text{​}}\gamma_{i}R_{i}O_{ea}+\;\sum^{\text{​}}\gamma_{i}F_{i}O_{ea}+\;\sum^{\text{​}}\gamma_{i}R_{i}F_{i}O_{ea}\;\;+\sum^{\text{​}}\gamma_{i}H^{\prime }{}_{b,i}+\sum^{\text{​}}\gamma_{i}L^{\prime }{}_{z}+\varepsilon_{i,2}$$

Based on empirical studies focusing on determinants of milk consumption, for example, References [69,82,83,84], and the available data within the LSMS, we included the following household level controls in our analysis: gender, age, marital status, and educational background of the household head, household’s living standard, number of young children below the age of seven, children between the age of seven and nine, older people (aged 65 and above), and remaining household members (between the age of 10 and 64). Household composition variables were included in the first stage *H^’^_a,i_* but not in the second stage *H^’^_b,i_*. In the second stage, we only accounted for the total household size. We also considered milk cow and milk goat ownership to control for the household’s direct access to milk. Milk can also be sourced from sheep and camel. However, none of the households in the sample held milking sheep and 1.29% of households in the sample had a camel, but these were exclusively Muslim households. We thus dropped sheep and camel ownership from the analyses as their contribution to milk intake was negligible. Finally, we added location level controls by incorporating the household’s residence (be it in a rural village, a small town, or a large town), the milk price to control for market accessibility, and a regional state dummy to adjust for possible variations in milk consumption and production across the regions due to differences in livestock population densities [8], tribal practices [85], and religious densities. A detailed description of the dependent and explanatory variables used in the models can be found in Supplementary Material (Table S1). More extended statistical analyses to test the robustness of our findings can be found in the Supplementary Material (Tables S2 to S5 and Figures S1 and S2).

## 3. Results

### 3.1. Descriptive Statistics 

**Figure 1:**
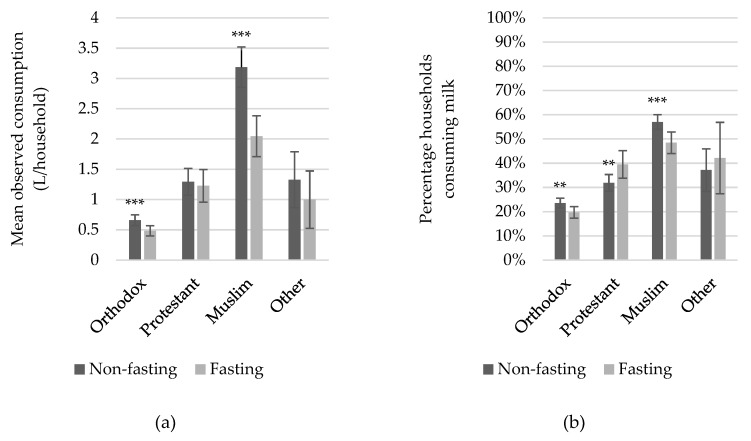
(**a**) Average observed milk consumption and (**b**) proportion of households consuming milk across religious groups during Orthodox fasting and non-fasting; with confidence intervals drawn at α = 0.05; *** and ** represent statistical significance at a probability of less than 1% and 5%, respectively.

When average milk consumption is calculated irrespective of a household’s religious affiliation, volumes consumed significantly decrease during Lent: from 1.55 liters/household during a non-fasting week recorded in the interviews compared to 1.04 liters/household during fasting. Figure 1 gives a comparison of the consumption patterns across the different religious groups (i.e., Orthodox, Protestant, Muslim, and other). Overall, Orthodox households seem to consume less milk compared to other religious communities (Figure 1a), which can partly be explained by the smaller proportion of Orthodox households that consume milk (Figure 1b). Figure 1a shows that Orthodox Christians consumed almost 30% less milk during Lent compared to their religious peers outside the fasting season (milk intake drops from 0.66 liters/household to 0.48 liters/household). This drop might seem small as we would expect consumption to drop to zero, yet, we also still found a large proportion of Orthodox households that consumed milk during Lent (Figure 1b). Comparing the household characteristics of Orthodox households that consumed milk during fasting with those that abstained from milk revealed that those households that maintained their milk consumption during Lent had significantly larger households with significantly more young children (on average, consuming households had twice as many young children). Moreover, milk cow herds of these households were twice as large as the herds of Orthodox households refraining from milk intake (Table 1). 

Moreover, it is not only Orthodox households that seem to reduce their consumption. The difference in milk consumption levels outside and during Lent across Muslim households was notable (Figure 1a). Figure 1b revealed that the proportion of Muslim households drinking milk reduced from 57% to 49%, which partially explained this sizable drop. An opposite trend was observed amongst Protestant households, although their total milk intake did not differ during versus outside Orthodox fasting. As explained in Section 2.3., consumption, availability, and sales of ASF alter sharply during fasting. If the market for dairy produce unravels during Orthodox fasts, we would expect that milk intake would drop for both Protestants and Muslims, but this was not what we observed. The limitations of our dataset do not allow us to further explore why we observed a differential impact on the Muslim and Protestant communities. 

**Figure 2:**
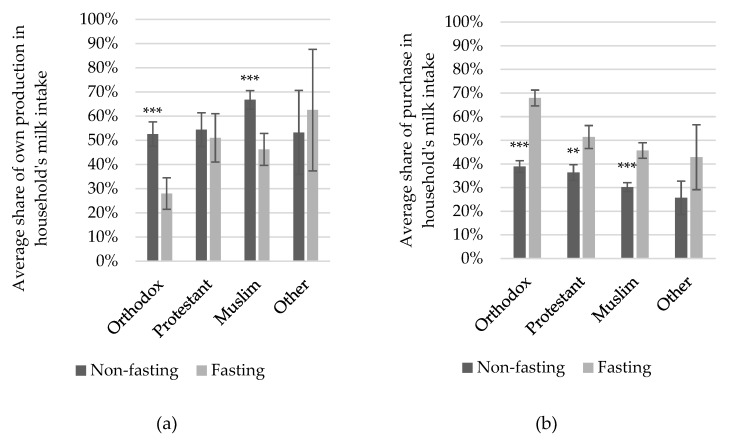
(**a**) Average share of home production and (**b**) purchase in total milk intake among milk-consuming religious households during Orthodox fasting and non-fasting; with confidence intervals drawn at α = 0.05; *** and ** represent statistical significance at a probability of less than 1% and 5%, respectively.

Next, we studied how fasting impacts milk sourcing strategies by reviewing the changes in the share of consumed milk coming from own milking cows or purchase during and outside fasting. We found that, on average, the share of home-produced milk in total milk intake lowered by a third for milk-drinking households interviewed during fasting (from 60% to 41%) while the share of purchase increases by 40% (from 32% to 53%). Figure 2 reveals that a lower sourcing from own milk production was only found amongst Orthodox and Muslim households interviewed during Lent compared to those interviewed outside Lent (Figure 2a), while the importance of milk purchases to satisfy the household’s milk needs increased significantly for Orthodox, Muslim, and Protestant households (Figure 2b). 

As milk prices are low during fasting, purchasing milk might be a better way to satisfy the household needs. For both Orthodox and Muslim households, total milk intake dropped during Orthodox fasting (Figure 1a), thus reducing the amount of milk to be sourced from either production or purchase. One may expect that both Orthodox and Muslim households might prefer to process the milk they produce into butter. This butter, when processed with proper sanitation methods and stored appropriately, can last for about a month [86] and hence be used as a coping strategy to overcome Orthodox fasting periods. Once the fasting period is finished, the butter might be sold or consumed by the household. However, we can argue that butter processing and sales are unlikely to fully cover the forgone milk consumption during fasting, nor will an increased reliance on milk purchase avoid an overproduction of milk during fasting. The first has been rebutted by Ayenew et al. [70], who found that butter sales are unaffected by Orthodox fasting, implying that butter sales do not go up once the Lent fasting period is finished (whereas this is true for the raw milk sales). Moreover, even if butter consumption might indeed increase after fasting, butter intake still remains rather low [69] and it is likely that during prolonged fasting periods, such as Lent, which last for about 55 days, the shelf life of butter is insufficiently long. Also, there is no increase in the volume of purchased milk, as Negassa [69] found that raw milk purchases decrease significantly during fasting compared to non-fasting periods. The limitations of our data set do not allow us to further explore these observations in greater depth.

### 3.2. Modelling the Probability of Milk Intake at the Household Level

Results from the Probit regression are presented in Table 2. The model yields a fairly high pseudo R^2^ of about 26%. Marginal effects of the factor variables included in the full Probit model (i.e., first column in Table 2) are presented in Table 3 and results from the Heckprobit regression can be found in Table 4 for the probability that part of the consumed milk was sourced from own production and in Table 5 for the probability that part of the consumed milk was retrieved through purchase. The first stage in the Heckprobit regressions corresponds to the full Probit model, as represented by the first column in Table 2. In the discussion below, we focused on the Orthodox, Protestant, and Muslim communities as the number of those with another religious affiliation was too small (*N* = 143) to draw non-fallacious conclusions.

Religion clearly impacts households’ milk intake as it altered the likelihood of a household to drink milk (Table 2). The effects are multiple and not limited to the Orthodox fasting community, and remain fairly constant when gradually building up the model. In the following, we will focus on the results from the full Probit model. First, religious affiliation had a statistically significant effect on the probability of households to consume milk. Protestant and Muslim households were 8 and 20 percentage points more likely to consume milk compared to Orthodox households, respectively (Table 3). Second, when we interacted religious affiliation with the interview period (whether consumption records were gathered during or outside fasting), we found that the predicted average probability of a household to consume milk during a Lent fasting week dropped by eight percentage points compared to a non-fasting week, irrespective of a household’s religious affiliation (Table 3). 

Although this drop might seem small, part of the households’ consumption data that were labelled as fasting reflected consumption both during Lent and non-fasting days, which might happen if a household was interviewed in the beginning of the Lent fasting period or after Lent fasting. When we detailed the impact of Orthodox fasting across the different religious groups, we found that the probability of milk intake decreased by 10 and 9 percentage points for Orthodox and Muslim households, respectively, while Protestant households’ probability increased by 3 percentage points (Table 3). Since the Orthodox and Muslim community jointly represent 80% of the Ethiopian population, this observation implies a non-negligible impact on national milk demand. Third, we found an effect of the milk cow herd size, along with religious affiliation and period of consumption. In the descriptive analysis, we observed significant differences in the sizes of milk cow herds for the different religious groups interviewed during and outside Lent. To control for this, we introduced a three-way interaction term of religious affiliation, interview period, and size of the milk cow herd. Larger herd sizes tended to have a positive effect on the probability of a household to consume milk, irrespective of fasting or non-fasting, for all religious denominations studied except for the Orthodox households (Table 2). Regardless of the number of milking cows that an Orthodox household possessed, fasting dramatically reduced their probability to consume milk (the coefficient is −0.16).

Next, we studied how fasting impacted on the milk market development by reviewing the changes in the likelihood to source consumed milk from own milking cows (Table 4) or purchase (Table 5) during and outside fasting. This allowed us to assess the societal impact in terms of milk market changes. Generally, we observed that Orthodox fasting negatively affected purchase intention, especially so for Orthodox (*p* = 0.12) and Protestant households, who were less likely to buy milk from the local market. This underscored the observation of unravelling markets during Orthodox fasting. This reduced likelihood to source milk from purchase was not compensated for by an increased reliance on home production, except for Protestant households. Furthermore, we found that in dominant Orthodox localities, the likelihood to buy milk decreased significantly, irrespective of religious affiliation. This suggests that due to repeating fasting spells, local dairy market development in such settings has been hampered. Only Muslim households were increasingly likely to source milk from purchase in dominant Orthodox settings. However, milk purchase volumes do not increase during fasting (see Negassa [69]), nor is milk increasingly sourced from own production (Table 4), hence this implies that milk intake decreases the more dominant the Orthodox religion is within a locality and thus that religious fasting practices are increasingly more likely to spill over to neighbours from another religious denomination (evidence also provided in Supplementary Material, Tables S2 and S3). 

## 4. Discussion

The results for all other controlling variables are as expected. The presence of young children and elderly people increased the likelihood of a household to drink milk by 3 and 5 percentage points for each additional young child and elderly person, respectively (Table 3). When the household head was female, and/or received secondary and above secondary school education, households were more inclined to consume milk (probability rose by 7 and 9 percentage points, respectively) (Table 3). Moreover, rising living standards and milk cow herd sizes positively affected the probability of milk intake (Table 2). Having one additional milking cow or a doubling of household nominal expenditure increased a household’s probability to consume milk by approximately 20 percentage points (Table 3). However, the effect of cow ownership reduced with increasing living standards (negative interaction), and vice-versa, which suggests that cows were particularly important for poor(er) households to provide access to milk (Table 4 first stage). This was supported by the results from the Heckprobit model, which revealed that mainly poor and low-educated households sourced their milk from home production, as suggested by the negative coefficients for both the income logarithm and educational background of the household head (Table 4). 

High milk prices were found to curtail the demand for milk (probability to consume dropped by three percentage points when milk prices increased by one Birr) (Table 3). This negative price elasticity illustrates that milk is a luxurious product that is mostly consumed by the better off households. The high price of milk is attributed to insufficient production and high marketing costs [74]. Moreover, Bachewe et al. [20] found that costs of ASF increase over time, reflected by the fact that during the period 2005–2011, expenditures on ASF grew by 22%, while actual ASF intake only slightly increased by about 6% [19]. 

Finally, location matters. We found that households in urban areas were more likely to consume milk and tended to rely more on local milk markets than on home milk production compared to their rural counterparts (suggested by the negative interaction term between milk price and location in the first stage of the Heckprobit regression in Table 4). Densely populated areas are known to ease the establishment of local markets as they face lower transaction costs [87]. The above observations were, however, only significant for small urban towns. Higher transaction costs may still persist in large urban areas, hampering the development of milk markets, which combined with the (more) limited space for keeping milking cows, poses difficulties in accessing milk for urban households [70].

## 5. Conclusions

Using country-wide data from the Living Standard Measurement Studies, we accentuated the so-far underexplored impact of religiously inspired consumption rituals. We studied the impact of fasting rituals amongst Orthodox households not only on consumption decisions, but also on the sourcing strategies of consumed milk. Including the consumption and sourcing patterns of households of different religious affiliations in the study allowed us to account for spillover effects across the society. We showed that Orthodox fasting reduced milk intake and altered sourcing strategies of consumed milk, both impacts which were not only limited to the Orthodox community. The surprisingly negative effect of Orthodox Lent on both the Muslim milk consumption and the propensity of Protestants and Muslims to consume milk from the local market (without an increased reliance on home production) when surrounded by an increasing number of Orthodox Christians is suggestive of the fact that the Orthodox reduction in milk consumption during Orthodox Lent spills over to the followers of other religious denominations. 

Our study herewith presents evidence that religious practices can have negative repercussions on the development of an effective functioning milk value chain. While factors such as income, urbanization and population are dynamic, religion is rather static and steadfast. We can thus expect that religious values and related fasting practices will persist in the near future. This study underscores the need to address demand-side inefficiencies and account for religiously inspired cultural differences in order to promote the development of agriculture and food production, notably the Ethiopian dairy sector in this specific case. Specifically, our results invite the policy question of how to mitigate the impact of Orthodox fasting on the dairy supply chain. Orthodox fasting lowered the demand for milk and milk products, which in turn adversely affected the sales opportunities of dairy producers and retailers. Below, we provide three examples of soft measures that may mitigate the impact of Orthodox fasting, in the belief that hard measures, such as the prohibition of fasting practices in consumption, are unlikely to be accepted by the Ethiopian society and are therefore bound to fail. First of all, dairy producers can try to cope with Orthodox fasting by overlapping cows’ dry periods with Orthodox fasting. However, given the multiple fasting periods within the Orthodox community that are scattered throughout the year, such a strategy may at best only partially tackle the demand–supply mismatch. Second, the government might create alternative market outlets for dairy products during fasting periods, for example by expanding school milk programs. Since children below the age of seven are exempt from fasting, such programs could be a very effective policy action not only to lower the demand–supply mismatch during Orthodox fasting, but also to improve food security and adequate nutrition amongst children. Finally, the government might invest or incentivize private investors to invest in strategies that structurally raise the shelf life of milk and milk products to overcome the fasting periods through the accumulation of a stock of milk produce. Specifically, efforts to stimulate the further development and adoption of dairy conservation and processing technologies are needed. To date, processing is largely small scale using traditional methods. Only 35 large-scale dairy processors are operating in the country (although often below their optimal capacity), and these are located principally around Addis Ababa. Moreover, it has been reported that during Orthodox fasting, these dairy processing plants reduce their supply of milk by requiring higher quality or paying lower prices to their milk suppliers [88].

Further research is imminent, which can also address the limitations that our study faces. More studies are required to assess the societal impact of religion on food systems by studying other religious practices and settings. Moreover, future studies should try to map through which pathways religion impacts various consumer groups to better understand why we observe a differential impact of Orthodox fasting on the Muslim and Protestant community. Also, the data we used face limitations. As we do not have information about households’ actual involvement in fasting rituals, we match dates of the fasting period (Lent) with the period of LSMS data collection to determine whether consumption data were gathered during or outside Orthodox fasting. Moreover, the households interviewed during fasting were not the same as those interviewed outside Lent. Therefore, we recommend that follow-up studies focus on a cohort of households before, during, and after fasting and incorporate information on households’ actual involvement in religiously inspired rituals, such as fasting.

## Figures and Tables

**Table 1:** Descriptive household characteristics of consuming and non-consuming Orthodox households during Lent.

	Mean (std. errors)	
	Non-Consuming Orthodox Households	Consuming Orthodox Households
Proportion of male heads	0.58 (0.02) ^a^	0.64 (0.03) ^a^
Age heads	43.17 (0.59) ^a^	41.72 (1.01) ^a^
Proportion of married heads	0.54 (0.02) ^a^	0.70 (0.03) ^b^
Proportion of heads with:		
Illiterate or informal education	0.40 (0.02) ^a^	0.30 (0.03) ^b^
Primary education	0.26 (0.02) ^a^	0.29 (0.03) ^a^
Secondary education	0.23 (0.02) ^a^	0.31 (0.03) ^b^
College/university education	0.10 (0.01) ^a^	0.10 (0.02) ^a^
Number of young children (0–6)	0.46 (0.03) ^a^	0.86 (0.06) ^b^
Number of children (7–9)	0.27 (0.02) ^a^	0.35 (0.04) ^a^
Number of elderly (65 and above)	0.17 (0.01) ^a^	0.12 (0.03) ^a^
Number of remaining members (10–64)	2.93 (0.07) ^a^	3.34 (0.13) ^b^
Milk cow herd size	0.20 (0.02) ^a^	0.44 (0.08) ^b^
Milk goat herd size	0.00 (0.00)	0 (0)
Income (Birr/week)	403.65 (11.92) ^a^	683.62 (35.54) ^b^
Proportion residing in:		
Rural area	0.32 (0.02) ^a^	0.26 (0.03) ^a^
Small town area (urban)	0.06 (0.01) ^a^	0.04 (0.01) ^a^
Large town area (urban)	0.62 (0.02) ^a^	0.70 (0.03) ^b^

^a,b^ Means within a row with different superscripts differ significantly (*p* < 0.05). Standard errors are given in brackets.

**Table 2:** Results from Probit models.

Independent Variable	Probit 1	Probit 2	Probit 3	Probit 4
Religion *(Orthodox omitted)*				
Protestant	0.07 (0.09)	0.24 (0.07) ***	0.08 (0.08)	0.22 (0.07) ***
Muslim	0.46 (0.08) ***	0.54 (0.06) ***	0.50 (0.07) ***	0.48 (0.06) ***
Other	0.29 (0.19)	0.47 (0.13) ***	0.28 (0.16) *	0.34 (0.16) **
Interaction (religion × interview period) *(Orthodox* × *nonfasting omitted)*				
Orthodox × fasting	−0.29 (0.07) ***	-	−0.34 (0.07) ***	-
Protestant × fasting	0.12 (0.12)	-	0.09 (0.11)	-
Muslim × fasting	−0.21 (0.09) **	-	−0.23 (0.08) ***	-
Other × fasting	−0.22 (0.33)	-	0.20 (0.27)	-
Interaction (religion × interview period × milk cow herd size) *(Orthodox* × *nonfasting omitted)*				
Protestant × nonfasting × milk cow herd size	0.04 (0.08)	-	-	−0.03 (0.08)
Muslim × nonfasting × milk cow herd size	0.17 (0.10) *	-	-	0.16 (0.10) *
Other × nonfasting × milk cow herd size	−0.01 (0.19)	-	-	−0.03 (0.19)
Orthodox × fasting × milk cow herd size	−0.16 (0.08) **	-	-	−0.28 (0.07) ***
Protestant × fasting × milk cow herd size	−0.03 (0.14)	-	-	−0.03 (0.13)
Muslim × fasting × milk cow herd size	0.11 (0.11)	-	-	−0.03 (0.10)
Other × fasting × milk cow herd size	1.56 (0.63) **	-	-	1.36 (0.58) **
Household controls	Yes	Yes	Yes	Yes
Location controls	Yes	Yes	Yes	Yes
Constant	−4.11 (0.35) ***	−4.30 (0.35) ***	−4.19 (0.35) ***	−4.20 (0.35) ***
				
Pseudo R^2^	0.27	0.26	0.26	0.26

***, **, and * represent statistical significance at a probability of less than 1%, 5%, and 10%, respectively. Standard errors are given between brackets.

**Table 3:** Predicted probability to consume milk at the household level evaluated at the mean of the covariates.

Independent Variable	Average Predicted Probability to Consume Milk
Religion *(Orthodox omitted)*	
Protestant	0.08 (0.02 ) ***
Muslim	0.20 (0.02) ***
Other	0.19 (0.06) ***
Interview period *(non-fasting omitted)*	
Fasting	−0.08 (0.02) ***
Interaction (religion × interview period)	
Orthodox × non-fasting	0.28 (0.02) ***
Orthodox × fasting	0.18 (0.02) ***
Protestant × non-fasting	0.31 (0.02) ***
Protestant × fasting	0.34 (0.03) ***
Muslim × non-fasting	0.47 (0.02) ***
Muslim × fasting	0.38 (0.03) ***
Other × non-fasting	0.38 (0.06) ***
Other × fasting	0.53 (0.11) ***
Sex head (*female omitted*)	−0.07 (0.02) ***
Age head	0.00 (0.00)
Marital status head (*not married omitted*)	0.01 (0.02)
Educational background head (*no or informal education omitted)*	
Primary education	−0.01 (0.02)
Secondary education	0.09 (0.03) ***
College/university education	0.09 (0.04) **
Number of young children (0–6)	0.03 (0.01) ***
Number of children (7–9)	0.02 (0.01)
Number of elderly (65 and above)	0.05 (0.02) **
Number of remaining members (10–64)	−0.02 (0.01) ***
Milk cow herd size	0.20 (0.02) ***
Milk goat herd size	0.02 (0.01) **
Logarithm of income	0.21 (0.01) ***
Location (*rural omitted*)	
Small town (urban)	−0.07 (0.03) **
Large town (urban)	0.09 (0.03) ***
Region	Yes
Milk price	−0.03 (0.01) ***

*** and ** represent statistical significance at a probability of less than 1% and 5%, respectively. Standard errors are given between brackets.

**Table 4:** Results from the Heckprobit model testing the likelihood to consume milk from own production.

Independent Variable	First Stage	Second Stage
Religion *(Orthodox omitted)*		
Protestant	0.06 (0.09)	−0.20 (0.27)
Muslim	0.44 (0.08) ***	−0.15 (0.28)
Other	0.30 (0.19)	−0.78 (0.37) **
Interaction (religion × interview period) *(Orthodox × non-fasting omitted)*		
Orthodox × fasting	−0.30 (0.07) ***	0.49 (0.52)
Protestant × fasting	0.12 (0.12)	0.64 (0.27) **
Muslim × fasting	−0.20 (0.09) **	−0.13 (0.14)
Other × fasting	−0.27 (0.33)	0.77 (0.80)
EA Orthodox concentration	-	0.38 (0.32)
Interaction (religion × EA Orthodox concentration) *(Orthodox omitted)*		
Protestant × EA Orthodox concentration	-	−0.61 (0.63)
Muslim × EA Orthodox concentration	-	−0.63 (0.56)
Other × EA Orthodox concentration	-	4.19 (3.10)
Interaction (interview period × EA Orthodox concentration) *(non-fasting omitted)*	-	−0.39 (0.61)
Interaction (religion × interview period × EA Orthodox concentration) *(Orthodox × fasting omitted)*		
Protestant × fasting × EA Orthodox concentration	-	0.84 (1.06)
Muslim × fasting × EA Orthodox concentration	-	0.15 (1.15)
Other × fasting × EA Orthodox concentration	-	−2.94 (3.57)
Interaction (religion × interview period × milk cow herd size) *(Orthodox × non-fasting omitted)*		
Protestant × non-fasting × milk cow herd size	0.05 (0.08)	-
Muslim × non-fasting × milk cow herd size	0.19 (0.10) *	-
Other × non-fasting × milk cow herd size	−0.02 (0.19)	-
Orthodox × fasting × milk cow herd size	−0.14 (0.08) *	-
Protestant × fasting × milk cow herd size	−0.01 (0.14)	-
Muslim × fasting × milk cow herd size	0.12 (0.11)	-
Other × fasting × milk cow herd size	1.65 (0.63) **	-
Sex head *(female omitted)*	−0.19 (0.06) ***	0.16 (0.12)
Age head	0.00 (0.00)	0.00 (0.00)
Marital status head *(not married omitted)*	0.03 (0.07)	0.11 (0.13)
Educational background head *(no or informal education omitted)*		
Primary education	−0.03 (0.06)	−0.27 (0.10) ***
Secondary education	0.24 (0.08) ***	−0.93 (0.16) ***
College/university education	0.25 (0.10) **	−0.96 (0.25) ***
Household size	-	0.10 (0.02) ***
Number of young children (0–6)	0.09 (0.02) ***	-
Number of children (7–9)	0.05 (0.04)	-
Number of elderly (65 and above)	0.12 (0.07) *	-
Number of remaining members (10–64)	−0.04 (0.01) ***	-
Milk cow herd size	1.62 (0.27) ***	1.10 (0.46) **
Milk goat herd size	0.06 (0.02) **	0.03 (0.02)
Logarithm of income	0.66 (0.04) ***	−0.17 (0.10)
Interaction (logarithm of income × milk cow herd size)	−0.20 (0.04) ***	−0.16 (0.07) **
Location *(rural omitted)*		
Small town (urban)	1.47 (0.63) **	0.16 (0.88)
Large town (urban)	0.08 (0.33)	−0.54 (0.74)
Region	Yes	Yes
Milk price	−0.08 (0.02) ***	0.13 (0.03) ***
Interaction (location × milk price) *(rural omitted)*		
Small town × milk price	−0.15 (0.05) ***	−0.08 (0.08)
Large town × milk price	0.01 (0.03)	−0.13 (0.06) **
Interaction (location × milk cow herd size) *(rural omitted)*		
Small town × milk cow herd size	0.24 (0.13) *	0.09 (0.12)
Large town × milk cow herd size	0.15 (0.19)	1.08 (0.32) ***
Constant	−4.12 (0.35) ***	−0.23 (0.91)
Athrho	−0.67 (0.26) ***	

***, **, and * represent statistical significance at a probability of less than 1%, 5%, and 10%, respectively. Standard errors are given between brackets. The dependent variable in the first stage regression is a dummy variable that takes on the value 1 if the household consumed milk in the last seven days prior to the interview and 0 otherwise. The dependent variable in the second stage regression is a dummy variable which takes on the value 1 if part of the milk consumed by the household originates from own production and 0 otherwise.

**Table 5:** Results from the Heckprobit model testing the likelihood to consume purchased milk.

Independent Variable	First Stage	Second Stage
Religion *(Orthodox omitted)*		
Protestant	0.06 (0.09)	−0.01 (0.29)
Muslim	0.46 (0.08) ***	−0.03 (0.29)
Other	0.33 (0.19) *	0.49 (0.39)
Interaction (religion × interview period) *(Orthodox × non-fasting omitted)*		
Orthodox × fasting	−0.30 (0.07) ***	−0.70 (0.45)
Protestant × fasting	0.12 (0.12)	−0.54 (0.28) *
Muslim × fasting	−0.22 (0.09) **	−0.07 (0.14)
Other × fasting	−0.27 (0.33)	−0.32 (0.87)
EA Orthodox concentration	-	−0.71 (0.34) **
Interaction (religion × EA Orthodox concentration) *(Orthodox omitted)*		
Protestant × EA Orthodox concentration	-	0.61 (0.63)
Muslim × EA Orthodox concentration	-	1.11 (0.55) **
Other × EA Orthodox concentration	-	−1.87 (1.70)
Interaction (interview period × EA Orthodox concentration) *(non-fasting omitted)*	-	0.88 (0.56)
Interaction (religion × interview period × EA Orthodox concentration) *(Orthodox × fasting omitted)*		
Protestant × fasting × EA Orthodox concentration	-	−0.12 (1.01)
Muslim × fasting × EA Orthodox concentration	-	−0.94 (0.89)
Other × fasting × EA Orthodox concentration	-	1.68 (3.13)
Interaction (religion × interview period × milk cow herd size) *(Orthodox × non-fasting omitted)*		
Protestant × non-fasting × milk cow herd size	0.06 (0.08)	-
Muslim × non-fasting × milk cow herd size	0.17 (0.10) *	-
Other × non-fasting × milk cow herd size	−0.04 (0.19)	-
Orthodox × fasting × milk cow herd size	−0.15 (0.08) *	-
Protestant × fasting × milk cow herd size	0.00 (0.14)	-
Muslim × fasting × milk cow herd size	0.13 (0.11)	-
Other × fasting × milk cow herd size	1.50 (0.65) **	-
Sex head *(female omitted)*	−0.20 (0.06) ***	−0.34 (0.12) ***
Age head	0.00 (0.00)	−0.00 (0.00)
Marital status head *(not married omitted)*	0.05 (0.07)	0.11 (0.13)
Educational background head *(no or informal education omitted)*		
Primary education	−0.03 (0.06)	0.36 (0.11) ***
Secondary education	0.25 (0.08) ***	0.72 (0.15) ***
College/university education	0.25 (0.10) **	0.78 (0.21) ***
Household size	-	−0.05 (0.02) **
Number of young children (0–6)	0.09 (0.02) ***	-
Number of children (7–9)	0.05 (0.04)	-
Number of elderly (65 and above)	0.14 (0.07) **	-
Number of remaining members (10–64)	−0.04 (0.01) ***	-
Milk cow herd size	1.62 (0.26) ***	−0.25 (0.49)
Milk goat herd size	0.05 (0.02) **	0.02 (0.02)
Logarithm of income	0.66 (0.04) ***	0.42 (0.10) ***
Interaction (logarithm of income × milk cow herd size)	−0.20 (0.04) ***	0.00 (0.08)
Location *(rural omitted)*		
Small town (urban)	1.20 (0.65) *	0.85 (1.18)
Large town (urban)	0.07 (0.33)	−1.19 (0.62) *
Region	Yes	Yes
Milk price	−0.08 (0.02) ***	−0.20 (0.03) ***
Interaction (location × milk price) *(rural omitted)*		
Small town × milk price	−0.12 (0.06) **	0.01 (0.10)
Large town × milk price	0.01 (0.03)	0.24 (0.05) ***
Interaction (location × milk cow herd size) *(rural omitted)*		
Small town × milk cow herd size	0.23 (0.13) *	−0.10 (0.13)
Large town × milk cow herd size	0.11 (0.19)	−0.41 (0.26)
Constant	−4.05 (0.35) ***	−0.55 (0.91)
Athrho	0.62 (0.25) **	

***, **, and * represent statistical significance at a probability of less than 1%, 5%, and 10%, respectively. Standard errors are given between brackets. The dependent variable in the first stage regression is a dummy variable that takes on the value 1 if the household consumed milk in the last seven days prior to the interview and 0 otherwise. The dependent variable in the second stage regression is a dummy variable that takes on the value 1 if part of the milk consumed by the household was sourced through purchase and 0 otherwise.

## Supplementary Materials

The following are available online at https://www.mdpi.com/2304-8158/8/5/167/s1. Table S1: Variables names, definitions, and description statistics. Table S2: Results from the extended Probit models accounting for the density of Orthodox believers in an enumeration area. Table S3: Results from the extended Probit models accounting for the density of churches in an enumeration area. Table S4: Results from the extended Heckprobit model accounting for the density of Orthodox believers in an enumeration area. Table S5: Results from the extended Heckprobit model accounting for the density of churches in an enumeration area. Figure S1: Predicted probability to consume milk at household level evaluated at the mean of the other covariates for different religious groups and interview periods at different densities of Orthodox believers in an enumeration area. Figure S2: Predicted probability to consume milk at household level evaluated at the mean of the other covariates for different religious groups and interview periods at different densities of churches in an enumeration area.
