# Supplementary material for: Religion, Food Choices, and Demand Seasonality: Evidence from the Ethiopian Milk Market
Source: Foods. 2019 May 16;8(5):167. doi: 10.3390/foods8050167 (PMC6560392; doi:10.3390/foods8050167)
Supplement: Supplementary file 1 [file foods-08-00167-s001.pdf]

Supplementary Material

Table S1. Variables names, definitions and description statistics.

Variable	Definition	Mean (std. errors)
Dependent variables		
Milk consumption	Dummy variable equal to 1 if a household consumed milk in the week prior to the interview; 0 otherwise	0.34 (0.01)
Consumed milk sourced from own production	Dummy variable equal to 1 if (part of) the milk consumed by the household originates from own production; 0 otherwise	0.55 (0.01)
Consumed milk sourced from purchase	Dummy variable equal to 1 if (part of) the milk consumed by the household is sourced through purchase; 0 otherwise	0.40 (0.01)
Independent variables		
Religion	Nominal variable equal to 1 if the religious affiliation of the household head is Orthodox, 2 for Protestant, 3 for Muslim, and 4 for other religious affiliations (e.g. Catholic, Pegan, Wakifata and traditional faiths); we assumed that the religious affiliation of the household head corresponds to the prevailing religious orientation within a household.	
	Proportion Orthodox households	0.50 (0.01)
	Proportion Protestant households	0.18 (0.01)
	Proportion Muslim households	0.29 (0.01)
	Proportion Other households	0.03 (0.00)
Interview period	As we have no information on whether or not a household participated in the Lent fasting, we matched the one week period of consumption data with the Lent period. This resulted in a dummy variable equal to 0 if records reflect consumption during nonfasting days, and 1 if (part of) the records were collected during fasting days.	0.34 (0.01)
EA Orthodox concentration	Continuous variable (ranging from 0 to 1) representing the share of Orthodox households relative to the other religions at enumeration area-level; this variable was calculated by dividing the number of interviewed Orthodox members by the total number of religious members (irrespective of religious affiliation), accounting for household size.	0.50 (0.01)
EA church concentration	Continuous variable (ranging from 0 to 1) representing the share of churches relative to the total number of churches and mosques at enumeration area-level	0.61 (0.01)
Household controls		
Sex head	Dummy variable equal to 1 if household head is male; 0 otherwise	0.70 (0.01)
Age head	Continuous variable reflecting the age of the household head (years)	44.15 (0.22)
Marital status head	Dummy variable equal to 1 if household head is married (monogamous or polygamous); 0 otherwise	0.68 (0.01)

Educational background head	Ordinal variable equal to 0 if household head is illiterate or received informal/non-regular education (e.g. Kuran education); 1 if household attended primary education; 2 for secondary education; and 3 for college/university education	
	Proportion illiterate or informal education	0.53 (0.01)
	Proportion primary education	0.28 (0.01)
	Proportion secondary education	0.14 (0.00)
	Proportion college/university education	0.06 (0.00)
Number of young children	Continuous variable representing the number of household members less or equal to 6 years old	0.88 (0.01)
Number of children	Continuous variable representing the number of household members between 7 and 9 years old	0.45 (0.01)
Number of elderly	Continuous variable representing the number of household members greater or equal to 65 years old	0.18 (0.01)
Number of remaining members	Continuous variable representing the number of household members greater than 9 years old and less or equal to 64 years old	3.31 (0.03)
Milk cow herd size	Continuous variable representing the number of female cows owned by the household and that gave milk during the past 12 months prior to the interview	0.38 (0.01)
Milk goat herd size	Continuous variable representing the number of female goats owned by the household and that gave milk during the past 12 months prior to the interview	0.20 (0.02)
Logarithm of income	Continuous variable representing the logarithm of the nominal weekly consumption of a household. It is used to proxy a household's living standard. The variable is included in the LSMS dataset and is calculated based on the consumption from three sources: food, nonfood purchases, and education expenses (Birr/week)	5.82 (0.01)
Credit	Dummy variable equal to 1 if someone within the household borrowed on credit during the past year prior to the interview; 0 otherwise	0.25 (0.01)
Location controls		
Location	Nominal variable equal to 0 if a household resides in a rural area,1 for urban small town area, and 2 for urban large town area	
	Proportion rural	0.63 (0.01)
	Proportion small town (urban)	0.09 (0.00)
	Proportion large town (urban)	0.28 (0.01)
Region	Nominal variable representing the regional state where a household resides	
	Proportion Tigray	0.12 (0.00)
	Proportion Afar	0.03 (0.00)
	Proportion Amhara	0.20 (0.01)
	Proportion Oromia	0.20 (0.01)
	Proportion Somali	0.06 (0.00)
	Proportion Benshagul Gumuz	0.02 (0.00)
	Proportion SNNP	0.23 (0.01)
	Proportion Gambelia	0.02 (0.00)

	Proportion Harari	0.03 (0.00)
	Proportion Addis Ababa	0.06 (0.00)
	Proportion Diredwa	0.04 (0.00)
Milk price	Continuous variable representing the prevailing milk price for a household. The variable was calculated using the approach adopted by LSMS. First, prices were calculated for those households that had purchased milk (amount paid/amount purchased). Then the price was set as the median price at the lowest geographical unit for which there were at least 10 price observations (Birr)	12.03 (0.03)
Distance to the nearest market	Continuous variable representing the distance of a household to the nearest market (in kilometers)	54.05 (0.70)

To test the robustness of our results, we ran more extended models for both the Probit and Heckprobit regressions. First of all, we ran additional models using church density instead of Orthodox density, to approximate the dominance of Christian congregations within each EA. Both variables (church density and Orthodox density within an EA) were alternatingly included in the Probit and Heckprobit regressions. Other important additions were the inclusion of extra control variables for household characteristics (e.g. age squared of the household head, the interaction term of age and gender of the household head, a more detailed categorization of household composition, and whether or not a household took credit in the past year) and location characteristics (e.g. the interaction term of region and milk cow herd size, the distance of a household's residence to the nearest market, the interaction term of distance to the market and milk cow herd size, and the three way interaction between location, milk price and milk cow herd size). The regression results can be found in the tables (Tables S2–S5) below.

Generally, we observe that the inclusion of EA church concentration (Tables S3 and S5) yielded more significant effects than when accounting for the density of Orthodox members in an EA (Tables S2 and S4). The church density variable represents all possible Christian congregations within an EA rather than only the Orthodox community. This implies that in dominant Christian settings, the impact of religion on milk intake decisions is bigger. Furthermore, we see that our results remain fairly robust when incorporating extra control variables, although the effects become smaller. Firstly, Muslim households are more likely to consume milk compared to Orthodox households. Secondly, Orthodox fasting reduces the likelihood to consume milk, irrespective of religious denomination (in table S2 the p value is 0.131). This effect is especially strong for Orthodox households suggested by the negative interaction between Orthodox affiliation, fasting and milk cow herd size. The impact of Orthodox fasting is also bigger for the Muslim community as compared to the Protestant households. Thirdly, modelling the probability to source consumed milk from own production in Table S4 and S5 reveals that in dominant Christian setting, the importance of home production to satisfy milk demand increases, irrespective of religious affiliation (this effect was not significant when accounting for the density of Orthodox members in an EA, see table S4). Especially Protestant households are less likely to source milk from home production with an increasing density of Orthodox members and churches, as the effect for Muslim households was not significant.

In the extended Probit model (which corresponds to Probit 7 in both Table S2 and S3), we also included the church and Orthodox density variable at enumeration area level, the results of which can be seen in Figure S1 and S2. An increasing density of Orthodox members and Christian churches negatively impacts milk intake decisions, *ceteris paribus*. This effect is however dependent on the interview period and the religious affiliation of the household head. Whereas an increasing density of Orthodox members reduces milk intake for all religions irrespective of fasting or non-fasting period (Figure S1), the impact of church concentration is mixed (Figure S2). As such, we observe that an increasing church concentration barely affects milk intake decisions of Orthodox households during fasting. Protestant households are increasingly likely to consume milk during non-Orthodox fasting periods with rising church concentration but an opposite trend is observed during fasting. Muslim households consume significantly less milk during with increasing church density irrespective whether it is an Orthodox fasting or non-fasting period.

Table S2. Results from the extended Probit models accounting for the density of Orthodox believers in an enumeration area.

Independent Variable	Probit 1	Probit 2	Probit 3	Probit 4	Probit 5	Probit 6	Probit 7
Religion *(Orthodox omitted)*							
Protestant	0.24 (0.07)***	0.07 (0.08)	0.07 (0.13)	0.07 (0.08)	-0.08 (0.14)	-0.04 (0.15)	-0.08 (0.16)
Muslim	0.54 (0.06)***	0.50 (0.07)***	0.36 (0.13)***	0.34 (0.08)***	0.31 (0.14)**	0.34 (0.15)**	0.34 (0.16)**
Other	0.45 (0.13)***	0.26 (0.16)	0.54 (0.20)***	0.30 (0.14)**	0.34 (0.21)	0.38 (0.23)	0.36 (0.25)
Interview period *(non-fasting omitted)*							
Fasting	-	-0.35 (0.07)***	-	-0.21 (0.05)***	-0.45 (0.16)***	-0.36 (0.23)	-0.34 (0.23)
Interaction (religion × interview period) *(Orthodox × non-fasting omitted)*							
Protestant × fasting	-	0.45 (0.12)***	-	-	0.53 (0.16)***	0.41 (0.27)	0.46 (0.28)
Muslim × fasting	-	0.11 (0.11)	-	-	0.23 (0.16)	0.12 (0.25)	0.13 (0.25)
Other × fasting	-	0.56 (0.28)**	-	-	0.79 (0.30)***	0.67 (0.43)	-0.00 (0.56)
EA Orthodox concentration			-0.37 (0.14)***	-0.39 (0.10)***	-0.37 (0.15)**	-0.33 (0.17)**	-0.35 (0.17)**
Interaction (religion × EA Orthodox concentration) *(Orthodox omitted)*							
Protestant × EA Orthodox concentration	-	-	0.14 (0.24)	-	0.01 (0.24)	-0.06 (0.30)	-0.00 (0.30)
Muslim × EA Orthodox concentration	-	-	-0.09 (0.23)	-	-0.12 (0.23)	-0.20 (0.29)	-0.17 (0.29)
Other × EA Orthodox concentration	-	-	-0.93 (0.45)**	-	-1.14 (0.46)**	-1.23 (0.60)**	-1.18 (0.60)*
Interaction (interview period × EA Orthodox concentration) *(non-fasting omitted)*							
Fasting × EA Orthodox concentration	-	-	-	-	0.10 (0.18)	-0.01 (0.27)	0.06 (0.27)
Interaction (religion × interview period × EA Orthodox concentration) *(Orthodox × non-fasting omitted)*							
Protestant × fasting × EA Orthodox concentration	-	-	-	-	-	0.20 (0.48)	0.09 (0.49)
Muslim × fasting × EA Orthodox concentration	-	-	-	-	-	0.20 (0.43)	0.13 (0.43)
Other × fasting × EA Orthodox concentration	-	-	-	-	-	0.22 (0.93)	1.05 (1.04)
Interaction (religion × interview period × milk cow herd size) *(Orthodox × non-fasting omitted)*							
Orthodox × fasting × milk cow herd size	-	-	-	-	-	-	-0.18 (0.08)**
Protestant × non-fasting × milk cow herd size	-	-	-	-	-	-	0.04 (0.11)
Protestant × fasting × milk cow herd size	-	-	-	-	-	-	-0.07 (0.16)
Muslim × non-fasting × milk cow herd size	-	-	-	-	-	-	-0.04 (0.11)
Muslim × fasting × milk cow herd size	-	-	-	-	-	-	-0.09 (0.13)
Other × non-fasting × milk cow herd size	-	-	-	-	-	-	-0.02 (0.21)
Other × fasting × milk cow herd size	-	-	-	-	-	-	1.43 (0.71)**
Household controls	Yes	Yes	Yes	Yes	Yes	Yes	Yes
Location controls	Yes	Yes	Yes	Yes	Yes	Yes	Yes
Constant	-4.74 (0.69)***	-4.59 (0.70)***	-4.37 (0.71)***	-4.29 (0.70)***	-4.22 (0.72)***	-4.26 (0.72)***	-4.16 (0.73)***
Pseudo R^2	0.27	0.28	0.27	0.28	0.28	0.28	0.28

***, **, and * represent statistical significance at a probability of less than 1%, 5%, and 10% respectively. Standard errors are given between brackets.

Table S3. Results from the extended Probit models accounting for the density of churches in an enumeration area.

Independent Variable	Probit 1	Probit 2	Probit 3	Probit 4	Probit 5	Probit 6	Probit 7
Religion *(Orthodox omitted)*							
Protestant	0.24 (0.07)***	0.07 (0.08)	0.08 (0.23)	0.28 (0.07)***	-0.18 (0.24)	-0.68 (0.32)**	-0.70 (0.32)**
Muslim	0.54 (0.06)***	0.50 (0.07)***	0.71 (0.11)***	0.45 (0.07)***	0.58 (0.13)***	0.53 (0.14)***	0.54 (0.14)***
Other	0.45 (0.13)***	0.26 (0.16)	0.95 (0.52)*	0.56 (0.15)***	0.64 (0.52)	0.97 (0.58)*	0.92 (0.60)
Interview period *(non-fasting omitted)*							
Fasting	-	-0.35 (0.07)***	-	-0.26 (0.05)***	-0.55 (0.14)***	-0.67 (0.17)***	-0.64 (0.17)***
Interaction (religion × interview period) *(Orthodox × non-fasting omitted)*							
Protestant × fasting	-	0.45 (0.12)***	-	-	0.42 (0.13)***	1.53 (0.47)***	1.56 (0.47)***
Muslim × fasting	-	0.11 (0.11)	-	-	0.27 (0.14)*	0.38 (0.20)*	0.36 (0.21)*
Other × fasting	-	0.56 (0.28)**	-	-	0.68 (0.31)**	-1.28 (1.39)	-0.45 (1.43)
EA church concentration	-	-	-0.02 (0.11)	-0.21 (0.09)**	-0.15 (0.13)	-0.21 (0.14)	-0.24 (0.14)*
Interaction (religion × EA church concentration) *(Orthodox omitted)*							
Protestant × EA church concentration	-	-	0.24 (0.27)	-	0.36 (0.27)	0.93 (0.36)**	0.93 (0.36)**
Muslim × EA church concentration	-	-	-0.69 (0.21)***	-	-0.67 (0.21)***	-0.60 (0.25)**	-0.58 (0.26)**
Other × EA church concentration	-	-	-0.45 (0.60)	-	-0.36 (0.60)	-0.76 (0.68)	-0.72 (0.69)
Interaction (interview period × EA church concentration) *(non-fasting omitted)*							
Fasting × EA church concentration	-	-	-	-	0.18 (0.17)	0.35 (0.22)	0.43 (0.22)**
Interaction (religion × interview period × EA church concentration) *(Orthodox × non-fasting omitted)*							
Protestant × fasting × EA church concentration	-	-	-	-	-	-1.32 (0.54)**	-1.42 (0.56)**
Muslim × fasting × EA church concentration	-	-	-	-	-	-0.16 (0.38)	-0.23 (0.39)
Other × fasting × EA church concentration	-	-	-	-	-	2.23 (1.56)	0.76 (1.67)
Interaction (religion × interview period × milk cow herd size) *(Orthodox × non-fasting omitted)*							
Orthodox × fasting × milk cow herd size	-	-	-	-	-	-	-0.22 (0.09)**
Protestant × non-fasting × milk cow herd size	-	-	-	-	-	-	0.08 (0.11)
Protestant × fasting × milk cow herd size	-	-	-	-	-	-	0.02 (0.17)
Muslim × non-fasting × milk cow herd size	-	-	-	-	-	-	-0.09 (0.12)
Muslim × fasting × milk cow herd size	-	-	-	-	-	-	-0.07 (0.13)
Other × non-fasting × milk cow herd size	-	-	-	-	-	-	0.05 (0.25)
Other × fasting × milk cow herd size	-	-	-	-	-	-	0.00 (0.00)
Household controls	Yes	Yes	Yes	Yes	Yes	Yes	Yes
Location controls	Yes	Yes	Yes	Yes	Yes	Yes	Yes
Constant	-4.74 (0.69)***	-4.59 (0.70)***	-4.88 (0.72)***	-4.56 (0.71)***	-4.58 (0.73)***	-4.53 (0.74)***	-4.42 (0.75)***
Pseudo R^2	0.27	0.28	0.27	0.27	0.28	0.28	0.28

***,**, and * represent statistical significance at a probability of less than 1%, 5%, and 10% respectively. Standard errors are given between brackets.

Table S4. Results from the extended Heckprobit model accounting for the density of Orthodox believers in an enumeration area.

Independent Variable	First Stage	Second Stage
Religion *(Orthodox omitted)*		
Protestant	-0.10 (0.16)	-0.02 (0.34)
Muslim	0.33 (0.16)**	0.07 (0.34)
Other	0.35 (0.25)	-0.87 (0.49)*
Interview period *(non-fasting omitted)*		
Fasting	-0.34 (0.23)	0.05 (0.70)
EA Orthodox concentration	-0.35 (0.17)**	0.37 (0.40)
Interaction (religion x interview period) *(Orthodox × non-fasting omitted)*		
Protestant × fasting	0.47 (0.28)*	0.50 (0.79)
Muslim × fasting	0.13 (0.25)	-0.11 (0.72)
Other × fasting	-0.00 (0.56)	0.50 (2.12)
Interaction (religion × EA Orthodox concentration) *(Orthodox omitted)*		
Protestant × EA Orthodox concentration	0.02 (0.30)	-1.34 (0.75)*
Muslim × EA Orthodox concentration	-0.18 (0.29)	-0.95 (0.64)
Other × EA Orthodox concentration	-1.17 (0.60)*	4.94 (4.24)
Interaction (interview period × EA Orthodox concentration) *(non-fasting omitted)*		
Fasting × EA Orthodox concentration	0.06 (0.27)	-0.31 (0.79)
Interaction (religion × interview period × EA Orthodox concentration) *(Orthodox × non-fasting omitted)*		
Protestant × fasting × EA Orthodox concentration	0.07 (0.49)	1.39 (1.32)
Muslim × fasting × EA Orthodox concentration	0.15 (0.43)	-0.14 (1.42)
Other × fasting × EA Orthodox concentration	1.05 (1.05)	-4.41 (5.67)
Interaction (religion × interview period × milk cow herd size) *(Orthodox × non-fasting omitted)*		
Orthodox × fasting × milk cow herd size	-0.17 (0.08)**	0.23 (0.24)
Protestant × non-fasting × milk cow herd size	0.04 (0.11)	0.08 (0.19)
Protestant × fasting × milk cow herd size	-0.06 (0.16)	0.22 (0.41)
Muslim × non-fasting × milk cow herd size	-0.04 (0.11)	0.11 (0.21)
Muslim × fasting × milk cow herd size	-0.09 (0.13)	-0.13 (0.22)
Other × non-fasting × milk cow herd size	-0.01 (0.21)	0.13 (0.29)
Other × fasting × milk cow herd size	1.43 (0.71)**	6.30 (2,040.13)
Sex head *(female omitted)*	-0.07 (0.14)	0.24 (0.13)*
Age head	-0.02 (0.02)	-0.01 (0.02)
Age head squared	0.26 (0.20)	0.22 (0.29)
Interaction (sex * age head) *(female omitted)*	-0.00 (0.00)	-
Marital status head *(not married omitted)*	0.03 (0.07)	-
Educational background head *(no or informal education omitted)*		
Primary education	-0.03 (0.06)	-0.34 (0.11)***
Secondary education	0.24 (0.08)***	-1.00 (0.21)***
College/university education	0.19 (0.10)*	-1.05 (0.33)***
Number of infants (<1)	0.26 (0.07)***	0.19 (0.13)
Number of young children (1-6)	0.05 (0.03)*	0.14 (0.05)***
Number of children (7-9)	0.04 (0.04)	-
Number of adolescents (10-19)	-0.05 (0.02)**	-
Number of elderly (65 and above)	0.16 (0.07)**	-
Number of remaining members (10-64)	-0.06 (0.02)***	0.08 (0.05)
Size of milk cow herd	1.64 (0.31)***	3.48 (0.91)***
Size of milk goat herd	0.05 (0.02)**	0.04 (0.03)*
Logarithm of income	0.69 (0.04)***	0.17 (0.22)
Interaction (logarithm of income × milk cow herd size)	-0.19 (0.05)***	-0.20 (0.10)*
Region	Yes	Yes
Location *(rural omitted)*		
Small town (urban)	1.16 (0.63)*	1.97 (1.15)*
Large town (urban)	-0.19 (0.34)	0.29 (1.11)
Milk price	-0.09 (0.02)***	0.22 (0.07)***
Interaction (location × milk price) *(rural omitted)*		
Small town × milk price	-0.12 (0.06)**	-0.24 (0.10)**
Large town × milk price	0.02 (0.03)	-0.20 (0.09)**
Interaction (region × milk cow herd size)	Yes	Yes
Interaction (location × milk cow herd size) *(rural omitted)*		
Small town × milk cow herd size	0.16 (0.15)	4.35 (2.97)
Large town × milk cow herd size	0.08 (0.21)	0.39 (3.27)
Distance nearest market	0.01 (0.00)***	0.00 (0.00)
Distance nearest market squared	-0.15 (0.03)***	-0.03 (0.07)

Interaction (milk cow herd size × distance nearest market)	-0.00 (0.00)***	-0.00 (0.00)**
Interaction (location × milk price × milk cow herd size)		
Rural × milk price × milk cow herd size	-	-0.18 (0.06)***
Small town × milk price × milk cow herd size	-	-0.54 (0.26)**
Large town × milk price × milk cow herd size	-	-0.13 (0.27)
Credit use	-	0.18 (0.11)*
Constant	-4.11 (0.73)***	-4.81 (1.77)***
Athrho	0.17 (0.53)	

***,**, and * represent statistical significance at a probability of less than 1%, 5%, and 10% respectively. Standard errors are given between brackets. The dependent variable in the first stage regression is a dummy variable which takes on the value 1 if the household consumed milk in the last seven days prior to the interview and 0 otherwise. The dependent variable in the second stage regression is a dummy variable which takes on the value 1 if (part of) the milk consumed by the household originates from own production and 0 otherwise.

Table S5. Results from the extended Heckprobit model accounting for the density of churches in an enumeration area.

Independent Variable	First Stage	Second Stage
Religion *(Orthodox omitted)*		
Protestant	-0.69 (0.32)**	1.42 (0.95)
Muslim	0.53 (0.14)***	0.15 (0.36)
Other	0.94 (0.60)	-3.13 (1.33)**
Interview period *(non-fasting omitted)*		
Fasting	-0.64 (0.17)***	0.17 (0.62)
EA church concentration	-0.24 (0.14)*	0.87 (0.32)***
Interaction (religion × interview period) *(Orthodox × non-fasting omitted)*		
Protestant × fasting	1.55 (0.47)***	-1.17 (1.41)
Muslim × fasting	0.36 (0.21)*	-0.26 (0.63)
Other × fasting	-0.48 (1.43)	-9.50 (12,347.67)
Interaction (religion × EA church concentration) *(Orthodox omitted)*		
Protestant × EA church concentration	0.92 (0.36)**	-2.30 (1.04)**
Muslim × EA church concentration	-0.60 (0.26)**	-0.62 (0.58)
Other × EA church concentration	-0.72 (0.69)	2.25 (1.46)
Interaction (interview period × EA church concentration) *(non-fasting omitted)*		
Fasting × EA church concentration	0.42 (0.22)*	-0.37 (0.71)
Interaction (religion × interview period × EA church concentration) *(Orthodox × non-fasting omitted)*		
Protestant × fasting × EA church concentration	-1.40 (0.56)**	2.29 (1.54)
Muslim × fasting × EA church concentration	-0.21 (0.39)	0.20 (1.24)
Other × fasting × EA church concentration	0.78 (1.67)	10.01 (12,347.67)
Interaction (religion × interview period × milk cow herd size) *(Orthodox × non-fasting omitted)*		
Orthodox × fasting × milk cow herd size	-0.22 (0.09)**	0.37 (0.28)
Protestant × non-fasting × milk cow herd size	0.07 (0.12)	0.16 (0.19)
Protestant × fasting × milk cow herd size	0.02 (0.17)	0.23 (0.41)
Muslim × non-fasting × milk cow herd size	-0.08 (0.12)	0.21 (0.21)
Muslim × fasting × milk cow herd size	-0.07 (0.13)	0.00 (0.23)
Other × non-fasting × milk cow herd size	0.04 (0.25)	0.64 (0.32)**
Other × fasting × milk cow herd size	6.12 (1,183.29)	7.57 (3,550.79)
Sex head *(female omitted)*	-0.09 (0.15)	0.21 (0.12)*
Age head	-0.02 (0.02)	-0.01 (0.02)
Age head squared	0.24 (0.20)	0.23 (0.29)
Interaction (sex × age head) *(female omitted)*	-0.00 (0.00)	-
Marital status head *(not married omitted)*	0.03 (0.07)	-
Educational background household head *(no or informal education omitted)*		
Primary education	-0.04 (0.06)	-0.31 (0.12)**
Secondary education	0.24 (0.08)***	-1.07 (0.18)***
College/university education	0.24 (0.11)**	-1.06 (0.30)***
Number of infants (<1)	0.22 (0.07)***	0.13 (0.14)
Number of young children (1-6)	0.05 (0.03)*	0.12 (0.06)**
Number of children (7-9)	0.06 (0.04)	-
Number of adolescents (10-19)	-0.02 (0.02)	-
Number of elderly (65 and above)	0.18 (0.08)**	-
Number of remaining members (10-64)	-0.06 (0.02)**	0.12 (0.05)**
Size of milk cow herd	1.72 (0.33)***	2.30 (1.28)*
Size of milk goat herd	0.04 (0.02)*	0.05 (0.03)
Logarithm of income	0.68 (0.04)***	-0.08 (0.22)
Interaction (logarithm of income × milk cow herd size)	-0.20 (0.05)***	-0.12 (0.13)
Region	Yes	Yes
Location *(rural omitted)*		
Small town (urban)	1.24 (0.64)*	1.44 (1.26)
Large town (urban)	-0.12 (0.36)	-0.01 (1.13)
Milk price	-0.09 (0.03)***	0.24 (0.06)***
Interaction (location × milk price) *(rural omitted)*		
Small town × milk price	-0.12 (0.06)**	-0.19 (0.12)*
Large town × milk price	0.03 (0.03)	-0.16 (0.09)*
Interaction (region × milk cow herd size)	Yes	Yes
Interaction (location × milk cow herd size) *(rural omitted)*		
Small town × milk cow herd size	0.21 (0.17)	3.93 (2.88)
Large town × milk cow herd size	-0.00 (0.20)	2.72 (3.35)
Distance nearest market	0.01 (0.00)***	-0.00 (0.00)
Distance nearest market squared	-0.13 (0.03)***	0.04 (0.07)
Interaction (milk cow herd size × distance nearest market)	-0.00 (0.00)***	-0.00 (0.00)**
Interaction (location × milk price × milk cow herd size)		
Rural × milk price × milk cow herd size		-0.13 (0.07)**

Small town × milk price × milk cow herd size		-0.46 (0.25)*
Large town × milk price × milk cow herd size		-0.27 (0.27)
Credit use		0.19 (0.11)*
Constant	-4.31 (0.74)***	-3.49 (2.44)
Athrho	-0.38 (0.59)	

***, **, and * represent statistical significance at a probability of less than 1%, 5%, and 10% respectively. Standard errors are given between brackets. The dependent variable in the first stage regression is a dummy variable which takes on the value 1 if the household consumed milk in the last seven days prior to the interview and 0 otherwise. The dependent variable in the second stage regression is a dummy variable which takes on the value 1 if (part of) the milk consumed by the household originates from own production and 0 otherwise.

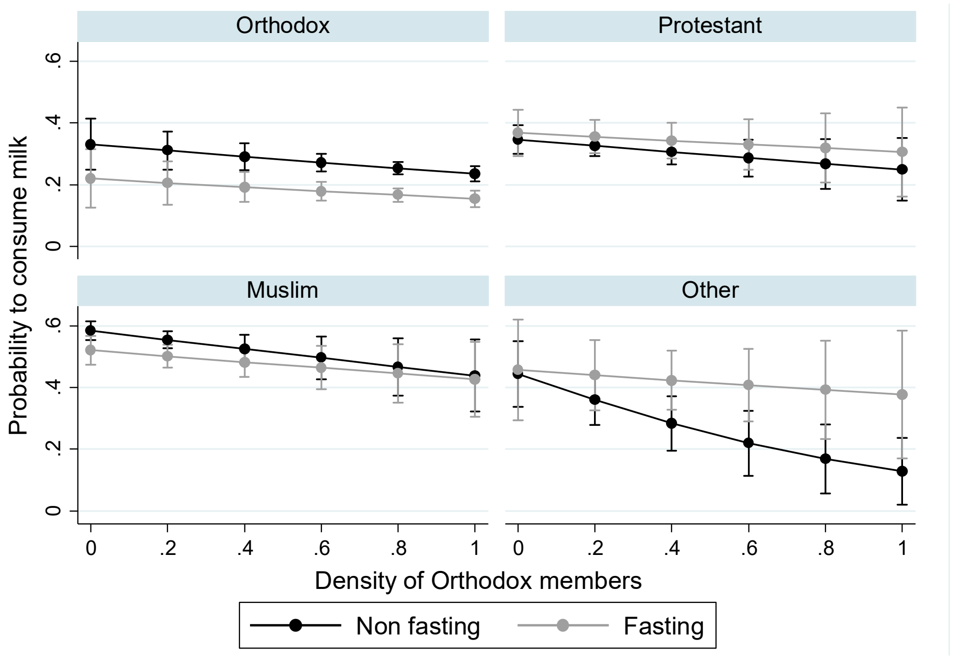

Figure S1. Predicted probability to consume milk at household level evaluated at the mean of the other covariates for different religious groups and interview periods at different densities of Orthodox believers in an enumeration area.

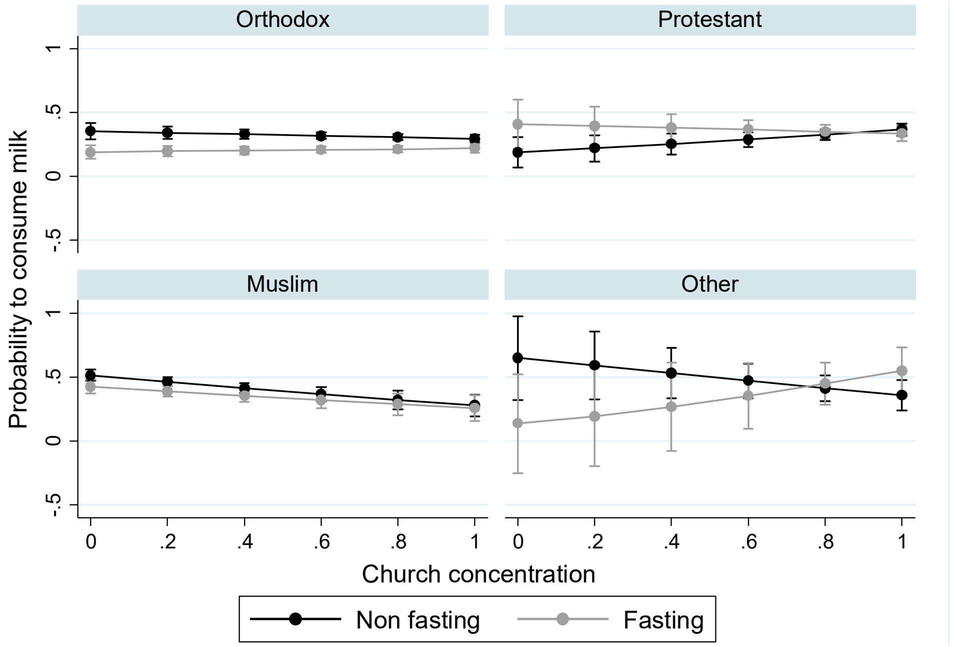

Figure S2. Predicted probability to consume milk at household level evaluated at the mean of the other covariates for different religious groups and interview periods at different densities of churches in an enumeration area.
